# Data-driven spatiotemporal modeling reveals personalized trajectories of cortical atrophy in Alzheimer’s disease

**Published:** 2025-11-12

**Authors:** Chunyan Li, Yutong Mao, Xiao Liu, Wenrui Hao

**Affiliations:** 1 Department of Mathematics, The Pennsylvania State University, University Park, PA, 16802, USA; 2 Department of Biomedical Engineering, The Pennsylvania State University, PA, 16802, USA; 3 Department of Biomedical Engineering, The Pennsylvania State University, PA, 16802, USA, Institute for Computational and Data Sciences, The Pennsylvania State University, PA, 16802, USA

**Keywords:** Alzheimer’s disease, spatiotemporal mathematical model, Alzheimer’s Disease Neuroimaging Initiative (ADNI), amyloid-*β*, tau, neurofunctional loss, sensitivity analysis

## Abstract

Alzheimer’s disease (AD) is characterized by the progressive spread of pathology across brain networks, yet forecasting this cascade at the individual level remains challenging. We present a personalized graph-based dynamical model that captures the spatiotemporal evolution of cortical atrophy from longitudinal MRI and PET data. The approach constructs individualized brain graphs and learns the dynamics driving regional neurodegeneration. Applied to 1,891 participants from the Alzheimer’s Disease Neuroimaging Initiative, the model accurately predicts key AD biomarkers—including amyloid-β, tau, neurodegeneration, and cognition—outperforming clinical and neuroimaging benchmarks. Patient-specific parameters reveal distinct progression subtypes and anticipate future cognitive decline more effectively than standard biomarkers. Sensitivity analysis highlights regional drivers of disease spread, reproducing known temporolimbic and frontal vulnerability patterns. This network-based digital-twin framework offers a quantitative, personalized paradigm for AD trajectory prediction, with implications for patient stratification, clinical trial design, and targeted therapeutic development.

## 1 Introduction

Alzheimer’s disease (AD) affects over 50 million people worldwide and represents the most common form of dementia, imposing a profound societal, economic, and personal burden [54]. Despite decades of intensive research and billions invested in therapeutic development, the underlying mechanisms that drive its onset and progression remain poorly understood, and no disease-modifying therapies have yet succeeded in the clinic. AD is characterized by extracellular amyloid-beta (Aβ) plaques and intracellular neurofibrillary tangles composed of hyperphosphorylated tau, which disrupt synaptic function, promote neuronal loss, and manifest as progressive cognitive decline pathogenesis [2, 24]. While the amyloid cascade hypothesis has provided a foundational framework, it fails to fully capture the multifactorial and dynamic nature of sporadic AD, which involves the interplay of amyloid and tau pathology, neuroinflammation, and other contributing biological processes.

Recent advances in biomarker imaging have transformed our ability to study AD in vivo. Positron emission tomography (PET) allows visualization of Aβ and tau deposition, structural MRI quantifies cortical atrophy, and cerebrospinal fluid (CSF) assays provide complementary molecular insights [15, 47]. Functional imaging further reveals network-level alterations linked to cognitive impairment [67], emphasizing that the critical questions are not only *what* changes occur in AD, but *how*, *where*, and *why* pathology propagates across the brain. These data create an unprecedented opportunity to study AD as a spatiotemporally dynamic disease, but also highlight the limitations of conventional analytical approaches.

Mathematical and computational modeling [3, 12, 26, 31, 33, 34, 38, 63] has emerged as an essential tool to integrate multi-modal data, formalize mechanistic hypotheses, and generate testable predictions and therapeutic interventions *in silico* [6, 14, 21, 46, 58]. Ordinary differential equation (ODE) models have been used to describe Aβ production, aggregation, and clearance [6, 8, 21], as well as tau hyperphosphorylation [7, 65]. Extensions incorporating neuroinflammation and other modulatory factors have provided additional mechanistic insights [43], and partial differential equation (PDE) frameworks capture the spatial propagation of pathology across brain regions [23, 48, 68]. Complementing these mechanistic approaches, machine learning and causal inference methods leverage large-scale clinical data to predict disease onset, stratify patients, and infer dynamic biomarker relationships [44, 66, 69, 70].

Despite these advances, critical gaps remain. Most models focus on individual pathways or global biomarkers, failing to capture the integrated spatiotemporal dynamics of amyloid, tau, and neuroinflammation [11, 51, 52, 59, 61]. Few frameworks provide personalized, spatially resolved predictions from longitudinal multi-modal imaging, limiting their translational relevance. Existing approaches often rely on MRI, regionally aggregated CSF or PET measures, lacking the spatial granularity needed to identify critical regions for targeted intervention [35, 50, 64].

Here, we present a novel, mechanistic, data-driven framework to model the spatiotemporal progression of AD biomarkers at the individual level. Our approach formulates a system of PDEs on the brain’s functional connectivity network, capturing the regional propagation of four key AD biomarkers: Aβ, tau, cortical atrophy, and functional network alterations. The model incorporates subject-specific parameters inferred from PET and structural MRI data, enabling personalized predictions while preserving mechanistic interpretability. Comprehensive parameter inference and two-level sensitivity analyses identify critical disease drivers and brain regions. To our knowledge, this is the first mechanistic spatiotemporal model integrating multiple clinically validated biomarkers in a patient-specific framework.

This framework provides three key advances:

1. It unifies temporal biomarker progression with spatial propagation across brain networks.
2. It enables patient-specific modeling of AD progression using multi-modal imaging biomarkers.
3. It facilitates the prediction of region-specific therapeutic responses, advancing precision medicine in AD.

By bridging mechanistic modeling, graph-based brain networks, and longitudinal neuroimaging, our approach provides a comprehensive, personalized view of AD progression, with direct implications for early diagnosis, individualized prognosis, and targeted therapeutic strategies.

## 2 Results

**Figure 1:**
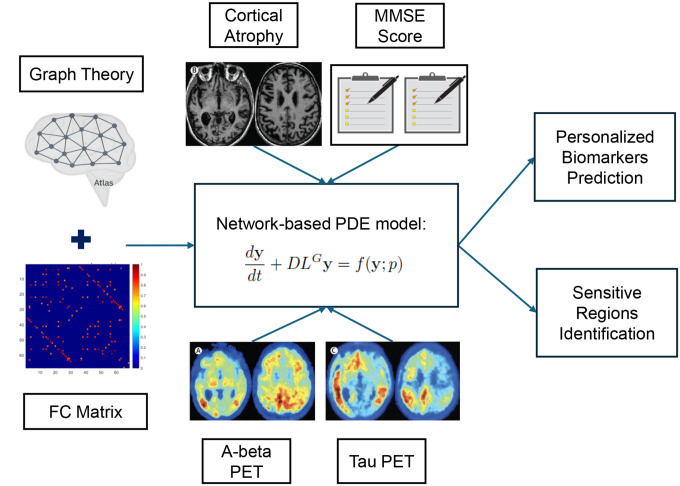
Workflow integrating graph theory, neuroimaging biomarkers (Aβ-PET, tau-PET, cortical atrophy, and MMSE score), AD pathophysiology, and mathematical modeling to construct a personalized digital twin of AD progression. The model, formulated as a spatiotemporal system of PDEs, captures biomarker diffusion and nonlinear interactions on the brain’s functional connectivity network. Coupled with sensitivity analysis, the framework enables personalized forecasting and identification of region-specific vulnerabilities with sensitivity analysis.

We applied the spatiotemporal generalized AD Biomarker Cascade (generalized ADBC) model to characterize individualized dynamics of clinically validated AD biomarkers and evaluate its potential for personalized disease forecasting. The model, governed by 14 parameters (6 global and 8 region-specific), captures heterogeneity across the brain’s functional network. Once estimated from neuroimaging data, it allows simulation of amyloid-beta (Aβ), tau, neurodegeneration (N), and cognitive decline (C), while a two-level sensitivity analysis identifies region-specific vulnerabilities. The overall workflow is illustrated in Figure 1, highlighting the model’s use for personalized biomarker prediction and sensitive-region identification.

### Individual-level simulations and predictions

**Figure 2:**
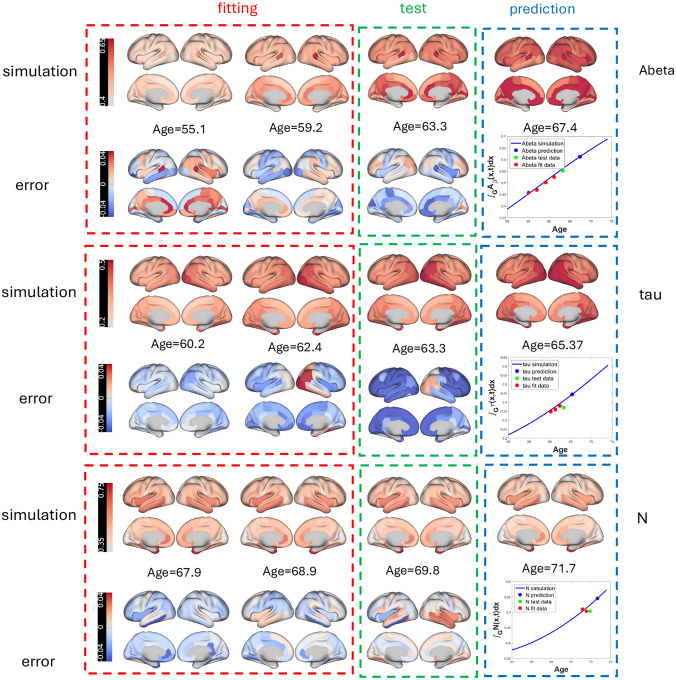
Model simulations of three spatially dependent biomarkers (Aβ, tau, N) for three individuals. Training fits, test evaluations, and future predictions are color-coded as red, green, and blue, respectively. Regional curves show accumulated biomarker levels across 68 brain regions.

Figure 2 demonstrates the model’s performance for three representative individuals. The model achieves prediction accuracies of 96.15%, 93.97%, and 93.35% for Aβ, tau, and N, respectively. Training fits (red rectangles), test evaluations (green), and future predictions (blue) are shown, with curves representing accumulated biomarker levels across 68 brain regions. By visualizing only two representative fitting points per biomarker, we simplify interpretation while preserving the spatial distribution of pathology. These results illustrate the model’s capability to forecast individualized biomarker trajectories, providing actionable information for clinicians to anticipate disease progression and personalize interventions.

### Population-level evaluation

To assess generalizability, we evaluated model performance across all subjects (Table 1). The nonhomogeneous (NH) model, which incorporates region-specific parameters, consistently outperforms the homogeneous (H) model in both training and testing, demonstrating improved adaptability to spatial heterogeneity. Mean testing accuracy gains are 1.87% (Aβ), 1.44% (tau), 4.40% (N), and 4.88% (C), with reduced standard deviations indicating enhanced robustness.

**Figure 3:**
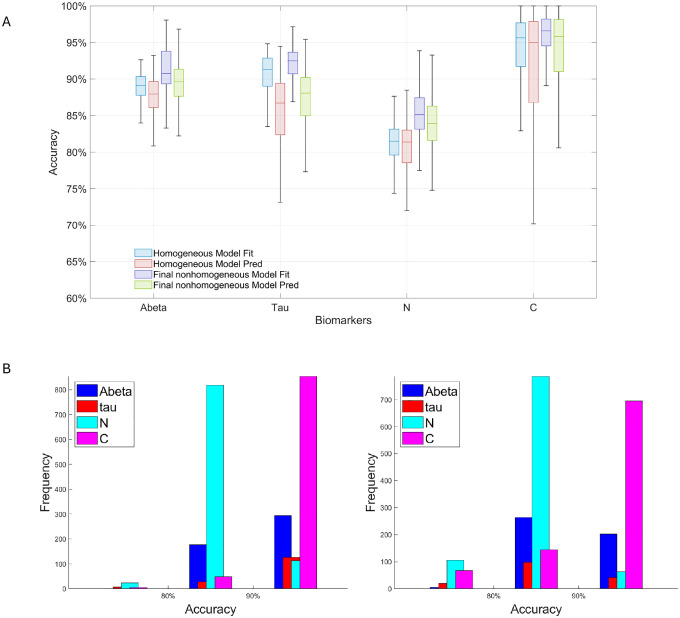
A: Boxplots of model accuracy across all subjects for H and NH models (interquartile range and median shown). B: Histograms of NH model fitting (left) and prediction (right) accuracies across all subjects and biomarkers.

Boxplots and histograms (Figure 3) further confirm that most subjects achieve over 80% accuracy across biomarkers under the NH model. Together, these results support the utility of incorporating regional heterogeneity for reliable, personalized predictions at the population level.

### Impact of training strategies

**Figure 4:**
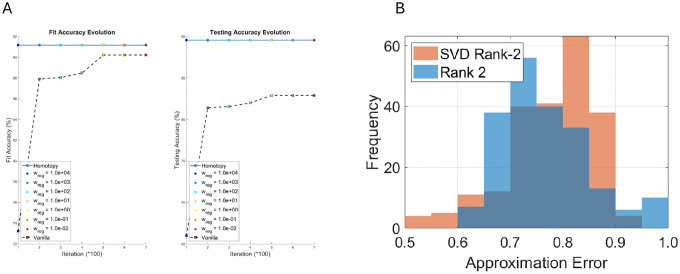
A: Comparison of homotopy regularization (solid line with circles) and vanilla training (dashed line with squares) across optimizer iterations. B: Relative error of rank-2 approximation of patient-specific FC matrix compared to SVD of true FC. Despite the full-rank difference, low-rank approximation achieves reasonable accuracy.

We next examined the influence of training methodology. For two randomly selected individuals, homotopy regularization improves performance relative to vanilla training (Figure 4A), particularly at intermediate regularization weights $\left(w_{\text{reg}}\right)$. Additionally, low-rank approximations of patient-specific functional connectivity (FC) matrices, learned solely from AD biomarkers, achieve comparable accuracy to rank-2 SVD approximations of the true FC matrices (Figure 4B). This finding demonstrates that the model can effectively leverage low-dimensional representations of network connectivity.

### Two-level sensitivity analysis

**Figure 5:**
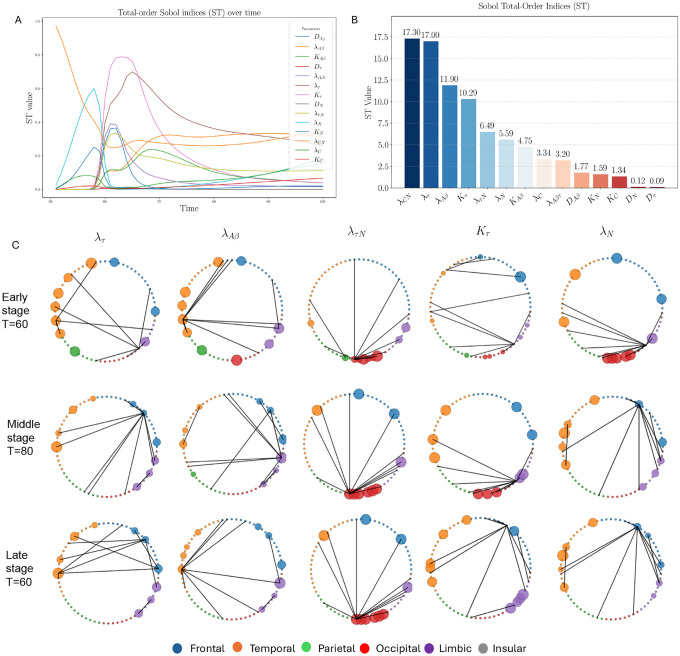
A: Dynamics of total sensitivity indices for 14 model parameters over age. B: Importance bar plot of sorted model parameters based on total sensitivity indices over age. C: The 68 Desikan–Killiany (DK) regions are grouped into six anatomical lobes. The second-level sensitivity analysis of key region-specific parameters is shown across three time stages (t=60,80, and 100). The first-order Sobol index $\left(S_{1}\right)$, represented by the circle radius, quantifies each lobe’s direct contribution to the model variance, while the second-order index ${(S}_{2})$, encoded by edge width, captures the strength of pairwise interactions between lobes. During computations, the model’s initial condition is set to the population mean at t=50, and the parameter ranges are defined by the minimum and maximum values of each parameter across the 68 regions. Each second-level sensitivity analysis is performed for a single region-specific parameter, with all other parameters fixed at the mean of the optimized values across all subjects.

We performed a two-level sensitivity analysis to identify critical model parameters and brain regions. First, assuming homogeneous parameters across all 68 DK regions, total sensitivity indices were calculated over age (Figure 5A), identifying six highly influential parameters: $\lambda_{CN},\lambda_{\tau },\lambda_{A_{\beta }},K_{\tau },\lambda_{\tau N}$, and $\lambda_{N}$ (Figure 5B). Second, we analyzed the top five region-specific parameters to pinpoint sensitive brain regions (Figure 5C), providing a quantitative basis for targeted monitoring and intervention.

Stage-wise sensitivity analysis reveals a dynamic network propagation mechanism: at early stages (t=60), first-order sensitivity indices $\left(S_{1}\right)$ are relatively uniform, with modest cross-lobe interactions $\left(S_{2}\right)$. By mid-stage (t=80), temporal–frontal, temporal–parietal, and temporal–limbic interactions dominate, while at late stage (t=100), inter-lobar interactions drive model sensitivity, with frontal–temporal and frontal–limbic couplings particularly prominent. These findings quantitatively support a network-based mechanism of disease spread, consistent with connectome-driven hypotheses.

Table 2 summarizes top DK regions for each biomarker and disease stage. The temporal and frontal lobes are most affected, with involvement intensifying over disease progression, whereas the insular lobe remains minimally affected. Aggregated across biomarkers, frontal and temporal lobes show increasing involvement from early to late stages, confirming that network hubs play a central role in AD progression [39].

## 3 Discussion

We present a region-specific, spatiotemporal model of AD progression that integrates biomarker dynamics within the ATN framework. By coupling PDEs on the brain functional-connectivity network with a two-level sensitivity analysis, we identified both key parameters and spatiotemporal patterns that govern the cascade from amyloid-β (Aβ) deposition to tau (τ) aggregation and subsequent neurodegeneration (N). This framework provides a mechanistic, patient-specific approach to capture the dynamics of AD biomarkers and their propagation across the brain.

### Model performance and predictive reliability

Leveraging multimodal neuroimaging data from the ADNI cohort, we parametrized the PDE model to construct individualized, region-specific biomarker trajectories. Unlike conventional AD criteria, which often overlook interindividual heterogeneity and focus on convergent phenotypes [32], our approach emphasizes personalized modeling as a pathway toward precision medicine.

The model demonstrated high accuracy in both fitting and prediction for Aβ,τ, N, and cognitive decline (Figure 3, Table 1), with consistently low variance between training and testing errors. Homotopy-regularized training improved convergence and optimization stability compared to standard training (Figure 4), mitigating overfitting in this high-dimensional parameter space. Furthermore, low-rank approximations of subject-specific FC matrices achieved comparable accuracy to SVD rank-2 approximations of true FC matrices, demonstrating that computational efficiency can be achieved without compromising predictive performance. Collectively, these results validate both the robustness and the predictive reliability of the model, supporting its use for patient-specific forecasting.

### Regional vulnerabilities and biological interpretation

The sensitivity analysis elucidated key parameters ${(\lambda }_{CN},\lambda_{\tau },\lambda_{A_{\beta }},K_{\tau },\lambda_{\tau N},\lambda_{N})$ that drive biomarker dynamics and highlighted spatiotemporal patterns of vulnerability [64]. Across disease stages, the temporal lobe consistently emerged as the earliest and most affected region, with frontal lobe involvement increasing in mid-to-late stages, whereas the insular lobe remained minimally affected and parietal/occipital lobes were relatively spared (Table 2). These patterns align with established neuropathological observations [10, 45, 57], wherein tau pathology initiates in the entorhinal and hippocampal cortices before propagating to temporal and frontal association areas. Imaging studies [20, 55] similarly report cortical thinning beginning in medial temporal regions and progressing anteriorly with disease severity. Our findings reinforce the temporal lobe as a neurodegenerative epicenter and the frontal lobe as a late-stage amplifier.

### Amyloid, tau, and neurodegeneration trajectories

The model recapitulates empirical biomarker propagation patterns observed in PET and MRI studies. Early Aβ accumulation was predicted in temporobasal and frontomedial cortices, followed by expansion to frontal and limbic regions [13, 30, 40]. Tau propagation persisted in temporal and frontal lobes across stages, consistent with functional-pathway-based stepwise spread [5, 41]. Neurodegeneration, reflected in cortical thinning, intensified primarily in frontal and temporal lobes during late stages, in agreement with longitudinal MRI studies [16, 53]. These trajectories capture the hallmark spatial hierarchy of AD progression: limbic–temporal initiation, frontal expansion, and relative parietal/occipital sparing.

### Network interactions and clinical implications

Second-order sensitivity analysis revealed that inter-lobe interactions dominate over intra-lobe effects, suggesting that AD spreads as a coordinated network disruption rather than isolated regional atrophy. This observation supports connectome-driven propagation frameworks [1, 9, 39, 60, 61], highlighting the role of functional connectivity in mediating cross-lobar pathology. By leveraging functional rather than purely structural connectivity, our model captures dynamic interactions that may underlie symptom evolution: frontal–temporal interplay could correspond to transitions from memory to executive dysfunction, while late insular involvement may relate to emotional and interoceptive deficits. These insights emphasize the potential for network-targeted therapeutic strategies that aim to preserve functional resilience rather than focusing solely on single regions.

### Limitations and future directions

Several limitations merit consideration. First, we assumed a static functional connectome, neglecting age- or disease-related network reconfigurations that could influence spatiotemporal spread. Second, uniform diffusion parameters $\left(D_{A_{\beta }},D_{\tau },D_{N}\right)$ may oversimplify subject-specific heterogeneity in amyloid, tau, and neuronal dysfunction.

Future work will incorporate dynamic functional networks whose topology evolves with disease progression and integrate multimodal calibration (MRI, PET, CSF biomarkers) for more comprehensive model fitting [4, 11, 29]. Parameter-uncertainty quantification and patient-specific optimization will enhance individualized predictions and enable virtual treatment simulations, including evaluation of pharmacological interventions on Aβ reduction and cognitive outcomes [36]. Finally, extending the PDE framework to include vascular, metabolic, and glial contributions could provide a more mechanistic understanding of the multifactorial drivers of neurodegeneration [27, 28, 42, 62].

## 4 Methods

### Data description

We accessed the multimodal data from the ADNI website following approval of our data use application (http://adni.loni.usc.edu/). The files titled “UC Berkeley - AV45 analysis [ADNI1,GO,2,3] (version:2020-05-12)” and “UC Berkeley - AV1451 analysis [ADNI1,GO,2,3] (version:2022-04-26)” compiled by ADNI were utilized to obtain Aβ-PET and tau-PET regional standardized uptake value ratios (SUVRs), Regional SUVRs were determined by dividing the standardized uptake values (SUVs) of the target regions by the SUV of the whole cerebellum, which served as the reference region because of its low specific binding and consistent uptake across the study population. In this study, we quantify neurodegeneration using cortical thickness, a well-established MRI-based biomarker that reflects neuronal loss and structural atrophy in Alzheimer’s disease (AD). This approach follows previous studies demonstrating that cortical thinning is strongly associated with AD pathology and predicts future cognitive decline [18, 19, 25, 37]. Specifically, reduced cortical thickness in AD-signature regions such as the medial temporal, inferior parietal, and posterior cingulate cortices has been shown to correlate with amyloid and tau burden as well as with disease progression. Cortical thickness data was obtained from ADNI as part of the “UCSF-Cross-Sectional FreeSurfer (6.0) [ADNI3]” and “UCSF-Cross-Sectional FreeSurfer (5.1) [ADNI1, GO, 2]” datasets. Functional connectivity (FC) data was derived by calculating Pearson’s correlation coefficients between time series extracted from regions defined by the DKT 68 atlas [17], based on rsfMRI data. All rsfMRI scans were acquired on 3 Tesla MR scanners across multiple ADNI participating sites, following a standardized protocol (https://adni.loni.usc.edu/data-samples/adni-data/neuroimaging/mri/mri-scanner-protocols/). The Mini-Mental State Examination (MMSE) scores were obtained from “Mini-Mental State Examination (MMSE) [ADNI1,GO,2,3,4]”. In this study, we did not impose a strict requirement for each subject to have data available for all modalities mentioned. Instead, our inclusion criterion focused on ensuring that each subject had data from at least three separate visits for one of the key measurements: tau-PET, amyloid-beta (Aβ-PET), or cortical thickness.

The study included 1,891 subjects from ADNI who were classified as cognitively normal (CN), mild cognitive impairment (MCI), AD, or of unknown status. The details are shown in Table 3.

### Data preparation

We normalize biomarker measurments across all subjects and brain subregions using:

(1) $$y_{j}^{i}=\frac{y_{j}^{i}}{y_{j}^{ref}}$$

where i=1,2,…,N indexes the i-th subject, j=1,2,…,68 denotes the j-th brain subregion. And $y_{j}^{\text{ref}}$ is the reference value for biomarker $y_{j}$ in the j-th region. Biomarkers y include amyloid-beta $\left(A_{\beta }\right)$, tau $\left(\tau_{\rho }\right)$, neurodegeneration (N) and cognitive impairment C.

To ensure uniform biomarker trajectories aligned with disease progression (all increasing with severity), we define linear transformations on the normalized biomarkers as follows:

- Amyloid β and tau proteins: both $A_{\beta }$ and $\tau_{\rho }$ increase with disease progression, as measured in PET imaging.
- Neurodegeneration: $N_{ct}$ quantified by cortical thickness which decreases as AD advances. We invert this trend by defining $N=1-N_{ct}$.
- Cognitive decline: Measured via the Mini-Mental State Examination (MMSE) score S, which declines with worsening pathology. We reverse its directionality using C=1−S.

This normalization and transformation framework ensures consistent biomarker behavior between subjects and subregions, simplifyingthe interpretation of the model.

### Patient-specific spatiotemporal partial differential equations model

**Figure 6:**
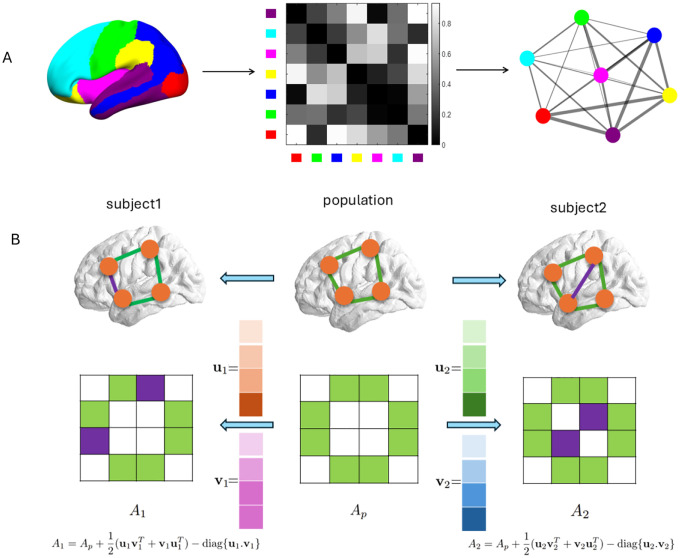
A: Topological representation of the human brain as a graph 𝒢=(𝒱,ℰ). Node set 𝒱 comprises 7 functionally defined brain regions (Left). Edge set ℰ is derived from a functional connectivity matrix, where connections between regions are thresholded for denoising (Middle). The undirected weighted graph 𝒢 visualizes connectivity strengths, with edge widths proportional to the magnitude of entries in the functional connectivity matrix (Right). This graph structure enables spatially resolved modeling of Alzheimer’s disease progression. B: A schematic representation of a simplified brain network as a graph, illustrating possible ways to derive a patient-specific adjacency matrix from the population-level adjacency matrix. The left arrow indicates that the topology remains unchanged, but the weights (FC values) on the edges differ, representing variations in the strength of functional connectivity between nodes. The right arrow illustrates a topology change, where a new edge is added to the graph, representing a functional connection strong enough to be included in the patient’s network.

We propose a spatially extended ADBC model that incorporates neuroimaging data and regional dynamics using graph theory and network science. This model can be used to capture the spatiotemporal progression of pathology in amyloid and taupathy, neuraldegenerion as well as cognitive decline. As illustrated in Figure 6A, we represent the brain’s geometry as an undirected weighted graph 𝒢=(𝒱,ℰ). Here, 68 nodes (vertices) correspond to DKT 68 atlas, while edges encode functional connectivity between regions. Edge weights, proportional to connectivity strength, are visualized as varying widths. This graph-based topology provides the foundation for our extended mathematical model, enabling spatially resolved modeling of disease progression while retaining the core dynamics of the ADBC framework.

Now we are ready to extend the ADBC model to be a spatial discretized partial differential equation (PDE) defined on the brain 𝒢 as follows:

(2) $$\frac{dA_{\beta }}{dt}+D_{A_{\beta }}L^{G}A_{\beta }=\lambda_{A_{\beta }}A_{\beta }\left(K_{A_{\beta }}-A_{\beta }\right)\;\frac{d\tau_{\rho }}{dt}+D_{\tau }L^{G}\tau =\lambda_{\tau_{\rho }A_{\beta }}A_{\beta }+\lambda_{\tau_{\rho }}\tau_{\rho }\left(K_{\tau_{\rho }}-\tau_{\rho }\right)\;\frac{dN}{dt}+D_{N}L^{G}N=\lambda_{N\tau_{\rho }}\tau_{\rho }+\lambda_{N}N\left(K_{N}-N\right)\;\frac{dC}{dt}=\lambda_{CN}\int_{G}Ndv+\lambda_{C}C\left(K_{C}-C\right)$$

The integration in last equation is defined as:

(3) $$\int_{G}N(v)dv=\frac{\sum_{v\in 𝒱}d_{v}N(v)}{\sum_{v\in 𝒱}d_{v}}$$

where $d_{v}$ is the degree of vertices v∈𝒱.

In the model, $A_{\beta }$ represents amyloid pathology, $\tau_{\rho }$ represents amyloid-related tau pathology (measured by tau-PET), N represents neuronal dysfunction/loss, and C represents cognitive impairment. $D_{A_{\beta }},D_{\tau },D_{N}\;\lambda_{CN},\lambda_{C}$ and $K_{C}$ are 6 scalar parameters and $\lambda_{A_{\beta }},\lambda_{\tau_{\rho }},\lambda_{N},\lambda_{\tau_{\rho }A_{\beta }}\;\lambda_{N\tau_{\rho }},K_{A_{\beta }},K_{\tau_{\rho }}$ and $K_{N}$ are 8 region-specific parameters which are graph functions 𝒱→R defined on 68 vertices. $D_{A_{\beta }},D_{\tau }$ and $D_{N}$ characterize the diffusion properties for $A_{\beta },\tau$ and N, respectively. $\lambda_{A_{\beta }},\lambda_{\tau_{\rho }}$ and $\lambda_{C}$ reflect the logistic growth rates of the various biomarker cascades. $\lambda_{\tau_{\rho }A_{\beta }}$ and $\lambda_{N\tau_{\rho }}$ and $\lambda_{CN}$ reflects linear growth rates of the biomarkers and determine the influence of various factors on the time-of-onset of the subsequent biomarker cascades. $K_{A_{\beta }},K_{\tau_{\rho }},K_{N}$ and $K_{C}$, represent the biomarker carrying capacities respectively. We would like to estimate these model parameters using the longitudinal neuroimaging data. $L^{G}$ is the patient-specific graph Laplacian corresponding to the patient’s specific brain functional connectivity network. Note that the graph Laplacian matrix is symmetric positive semi-definite which can be treated as the discretize version of −Δ using finite difference method with Neumann or periodic boundary condition. We will discuss how to learn patient specific graph Laplacian $L^{G}$ from limited functional connectivity data below.

### Learning patient-specific functional connectivity matrix

The graph Laplacian $L^{G}$ of a undirected weighted graph 𝒢=(𝒱,ℰ) is defined as the difference between the corresponding degree matrix D and adjacency matrix A

(4) $$L^{G}=D-A$$

where $A=\left(a_{ij}\right)\in R^{68\times 68}$, the adjacency matrix which is symmetric, is collection of weights $a_{ij}$ assigned for connected node pairs $\left(n_{i},n_{j}\right)\in ℰ$.

The corresponding degree matrix D that is, the number of edges attached to each node defined as follow:

(5) $$D_{ij}=\left\{\begin{array}{ll} \sum_{k=1}^{68}a_{ik}, & \text{if}\;i=j\\ 0 & \text{if}\;i\neq j \end{array}\right.$$

We use the functional connectivity (FC) matrix of 68 regions to compute the adjacency matrix A of brain graph 𝒢 after statistical truncation for denoising purpose. More precisely, we treat the two subregions are connected where the corresponding Pearson correlation coefficient value in FC matrix exceed 0.75 and the corresponding p value less than 1e−5. Consequently, an edge is established between such pairs of nodes in the graph representation of 𝒢. The corresponding adjacency matrix is weighted by the functional connectivity values after removing self-connections.

FC data was derived by calculating Pearson’s correlation coefficients between time series extracted from regions defined by the DKT 68 atlas [17], based on resting-state fMRI data. fMRI scans require expensive equipment and technical expertise. Conducting repeated scans over time (longitudinal studies) adds significantly to the cost. fMRI generates large datasets, espencially when acquired longitudinally. Processing these datasets to extract meaningful informations requires substantial computational resources. The size of fMRI datasets necessitates significant storage capacity. Patient data must be handled in compliance with strict privacy regulations, adding to the cost and complexity. Hence, a novel mathematical model/method for learning patient specific FC matrix (therefore Graph Laplacian) from limited FC data collected from public dataset is necessary.

We propose a qualitative method to learn a patient-specific adjacency matrix (FC matrix), defined as:

(6) $$A=A_{p}+\frac{1}{2}\left(uv^{T}+{vu}^{T}\right)-\text{diag}\left\{u.v\right\},$$

where u.v represents the elementwise product of u and v, resulting in a vector. The term $A_{p}=\frac{1}{K}\sum_{k=1}^{K}A^{\left(k\right)}$ denotes the population-level adjacency matrix derived from a limited population group. The rank-two matrix $\frac{1}{2}\left({uv}^{T}+{vu}^{T}\right)$ approximates the difference between a specific patient’s connectivity and the population mean, while the diagonal term −diag{u.v} ensures self-connections are removed. The parameters u and v, which are unit column vectors in $R^{68}$, quantify the patient-specific functional connectivity variation between different node pairs. The unit constraints $\|u{\|}_{2}=\|v{\|}_{2}=1$ are imposed to enhance numerical stability and prevent stiffness in solving ODE problems.

If u=v, then, (6) degenerate to the case with rank one approximation as follows:

(7) $$A=A_{p}+uu^{T}-\text{diag}\left\{u^{2}\right\}.$$

We propose using a rank-two matrix instead of a rank-one matrix to improve expressive capability and to address limitations associated with rank-one representations. A rank-one matrix, such as ${uu}^{T}$ with the constraint $\|u{\|}_{2}=1$, introduces unnecessary coupling between weights corresponding to different node pairs $\left(n_{i},n_{j}\right)$ and $\left(n_{k},n_{l}\right)$. For example:

(8) $${uu}^{T}=\left[\begin{array}{ccccc} u_{1}^{2} & u_{1}u_{2} & u_{1}u_{3} & \cdots & u_{1}u_{n}\\ u_{2}u_{1} & u_{2}^{2} & u_{2}u_{3} & \cdots & u_{2}u_{n}\\ \vdots & \vdots & \vdots & \ddots & \vdots \\ u_{n}u_{1} & u_{n}u_{2} & u_{n}u_{3} & \cdots & u_{n}^{2} \end{array}\right],$$

with the constraint $u_{1}^{2}+u_{2}^{2}+\cdots +u_{n}^{2}=1$.

In contrast, the rank-two representation ${uv}^{T}+{vu}^{T}$ provides greater flexibility:

(9) $${uv}^{T}+{vu}^{T}=\left[\begin{array}{cccc} u_{1}v_{1}+v_{1}u_{1} & u_{1}v_{2}+v_{1}u_{2} & \cdots & u_{1}v_{n}+v_{1}u_{n}\\ u_{2}v_{1}+v_{2}u_{1} & u_{2}v_{2}+v_{2}u_{2} & \cdots & u_{2}v_{n}+v_{2}u_{n}\\ \vdots & \vdots & \ddots & \vdots \\ u_{n}v_{1}+v_{n}u_{1} & u_{n}v_{2}+v_{n}u_{2} & \cdots & u_{n}v_{n}+v_{n}u_{n} \end{array}\right],$$

where the constraints $u_{1}^{2}+u_{2}^{2}+\cdots +u_{n}^{2}=1$ and $v_{1}^{2}+v_{2}^{2}+\cdots +v_{n}^{2}=1$ hold.

This representation offers significantly enhanced expressive capability compared to ${uu}^{T}$. For instance, in the rank-one matrix ${uu}^{T}$, the weight for the connection between nodes $n_{1}$ and $n_{2}\left(u_{1}u_{2}\right)$ is tightly constrained by the weight for the connection between $n_{5}$ and $n_{6}\left(u_{5}u_{6}\right)$ due to the unit norm constraint $\|u{\|}_{2}=1$. In reality, the strength of the connection between $n_{1}$ and $n_{2}$ should not inherently depend on the strength of the connection between $n_{5}$ and $n_{6}$. The rank-two representation ${uv}^{T}+{vu}^{T}$ overcomes this limitation, enabling a more accurate and flexible modeling of patient-specific FC.

As illustrated in Figure 6B, the brain network is represented as a graph with four nodes. The middle panel represents the population-level brain network, while individual deviations from this representation can be categorized into two fundamental cases:

- *edge weight changes:* The topology remains unchanged, but the weights on the exit edges differ from the population adjacency matrix. There exits an edge $e_{i}$ between nodes $n_{k}$ and $n_{h}$, and the weight is modified as $a_{kh}+\frac{1}{2}\left(u_{k}v_{h}+v_{k}u_{h}\right)-\delta_{kh}u_{k}v_{h}$ with constraint $-a_{kh}\leq \frac{1}{2}\left(u_{k}v_{h}+v_{k}u_{h}\right)-\delta_{kh}u_{k}v_{h}\leq 1-a_{kh}$. Note that the entries of u,v can be negative values as long as they satisfy constraint so that the weight valued could be increased or decreased.
- *topology changes:* New edges are added, representing additional functional connectivity between regions. A new edge $e_{i}^{\text{new}}$ is lighted up between nodes $n_{l}$ and $n_{m}$ with the associated weight $a_{lm}=\frac{1}{2}\left(u_{k}v_{h}+\right.\left.v_{k}u_{h}\right)-\delta_{kh}u_{k}v_{h}$ with constraint $0\leq \frac{1}{2}\left(u_{k}v_{h}+v_{k}u_{h}\right)-\delta_{kh}u_{k}v_{h}\leq 1$.

Luckily, the expression (6) can cover both cases. As shown in Figure 6B: (a) The left arrow indicates that the topology is identical to the population network, but the strength of functional connectivity (edge weights) varies indicated by a purple color of edge. (b) The right arrow illustrates a change in topology, where a new edge appears, representing functional connectivity between two regions strong enough to warrant inclusion. With this model, we address the challenges of data collection and computational expense, enabling efficient analysis of patient-specific brain networks. Then, with the definition of graph Laplacian (4) and the assumption for adjacency matrix (6), one can derive the patient specific Graph Laplacian is defined as

(10) $$L^{G}=L_{p}^{G}+\frac{1}{2}\text{diag}\left(u\cdot \left(v^{T}1\right)+v\cdot \left(u^{T}1\right)\right)-\frac{1}{2}\left(uv^{T}+{vu}^{T}\right)$$

We assume that the graph Laplacian is fixed in lifespan of a patient. $L_{p}^{G}$ denotes the population Graph Laplacian $L_{p}^{G}=\frac{1}{K}\sum_{k=1}^{K}\left(D^{\left(k\right)}-A^{\left(k\right)}\right)-=\frac{1}{K}\sum_{k=1}^{K}D^{\left(k\right)}-A_{p}$. Note that we use $A^{\left(k\right)}=\frac{A^{(k)T}+A^{\left(k\right)}}{2}$ to guarantee the symmetry which might be destroyed by the measurement noise.

### Parameters inference by novel hierarchical structured training strategy

Let $y={\left(A_{\beta },\tau_{\rho },N,C\right)}^{T}$ represents the biomarker vector obtained by solving model (2) and θ denotes the collection of all model parameters, and $y_{0}$ be the initial values of this model. $\tilde{y}\left(t_{i}\right)$ is the clinical data of a specific subject for given age $t_{i}$ and $y\left(t_{i};\theta \right)$ is the biomarker solution of the generalized ADBC model. The model parameters for each patient can be inferred by solving the constrained optimization problem:

(11) $$\underset{\theta ,y_{0},u,v}{\text{min}}ℒ\left(\theta ,y_{0},u,v\right)≔\sum_{i=1}^{M}\frac{{\left\|y\left(t_{i};\theta \right)-\tilde{y}\left(t_{i}\right)\right\|}_{2}^{2}}{{\left\|\tilde{y}\left(t_{i}\right)\right\|}_{2}^{2}}+w\|y(100;\theta )-1{\|}_{2}^{2}\;\text{subject}\;\text{to}:\;0\leq a_{kh}+\frac{1}{2}\left(u_{k}v_{h}+v_{k}u_{h}\right)-\delta_{kh}u_{k}v_{h}\leq 1\;\forall k,h=1,\ldots ,68.$$

where $u=\left(u_{1},\ldots ,u_{68}\right),v=\left(v_{1},\ldots ,v_{68}\right)$ are two unit vectors to learn patient-specific graph Laplacian, $a_{kh}$ is the (k,h) element of adjacency matrix $A_{p}.y_{0}$ is imposed as an optimized variable to avoid the overfitting of the measurement noise in ${\tilde{y}}_{0}$.

This optimization problem is to minimize the L2 loss on given clinical patient data points and constraint on y(100;θ)=1 as the penalty term which is used to ensure the biomarkers increasing with severity. This optimization problem was solved for each subject in the cohort using all available biomarker time points $t_{i},i=1,\ldots ,M$.

To address this challenging high-dimensional problem, we introduce a novel hierarchical structured training strategy incorporating a homotopy regularization technique to enhance the stability and efficiency of parameter learning. This hierarchical strategy consists of four sequential stages, where the solution obtained at each stage serves as the initial condition for the next, enabling a progressively refined learning of the extended ADBC model from neuroimaging data. To illustrate this procedure concretely, we take the Aβ equation as an example and describe the hierarchical approach in detail below. The equations for other biomarkers can be solved in an equation-by-equation manner [22].

1. **Homogenized Model:** To get a good initial guess for model parameters θ, we homogenize the model by homogenizing the regional variability. Namely, let $\left(D_{A_{\beta }}^{s},\lambda_{A_{\beta }}^{s},K_{A_{\beta }}^{s}\right)$ and $A_{\beta 0}^{s}$ be scalars. Then, the model parameters of this homogeneized model is $\theta_{A_{\beta }}^{s}=\left(I^{68}D_{A_{\beta }}^{s},I^{68}\lambda_{A_{\beta }}^{s},I^{68}K_{A_{\beta }}^{s}\right)$. Because different patients have different onset times, in order to give the initial conditions, we assume that the initial time for all people is 50 years old, and the corresponding initial value $I^{68}A_{\beta 0}^{s}$ is a parameter that needs to be inferred. The optimization problem is formulated as:
(12) $$\underset{\theta_{A_{\beta }},A_{\beta 0}}{\text{min}\;}ℒ\left(\theta_{A_{\beta }},A_{\beta 0}\right)=\sum_{i=1}^{M_{A_{\beta }}}\frac{{\left\|A_{\beta }\left(t_{i};\theta_{A_{\beta }}\right)-{\tilde{A}}_{\beta_{i}}\right\|}_{2}^{2}}{{\left\|{\tilde{A}}_{\beta_{i}}\right\|}_{2}^{2}}+w{\left\|A_{\beta }\left(100;\theta_{A_{\beta }}\right)-1\right\|}_{2}^{2},$$
  where coefficient w for the penalty term enforce a sigmoid-like solution. The parameters $\theta_{A_{\beta }}^{s}$ and $A_{\beta 0}^{s}$ are optimized using MATLAB’s fmincon iteratively with randomly generated initial guess with bounds $D_{A_{\beta }}\in [0,2]$, $\lambda_{A_{\beta }}\in [0,2]$, $K_{A_{\beta }}\in [1,2]$, and $A_{\beta 0}^{s}\in [0,1]$. And the initial condition of this equation is $I^{68}A_{\beta 0}^{s}≔A_{\beta }\left(t_{0}=50\right)$ with $I^{68}=\text{ones}(68,1)$. The spatial discreized PDE model is solved by ODE45.
2. **Non-homogenized model:** Extend homogenized parameters $\theta_{A_{\beta }}^{s}$ to non-homogenized parameters, denoted as $\theta_{A_{\beta }}^{68D}$ to account for regional variability. Let the sparse deviations from the scalar parameters denoted as $\epsilon_{A_{\beta }}=\theta_{A_{\beta }}^{68D}-\theta_{A_{\beta }}^{s}$, the optimization problem is formulated as:
(13) $$\underset{\epsilon_{A_{\beta }}}{\text{min}\;}ℒ\left(\epsilon_{A_{\beta }}\right)=\sum_{i}\frac{{\left\|A_{\beta }\left(t_{i};\epsilon_{A_{\beta }}+\theta_{A_{\beta }}^{s}\right)-A_{\beta }\left(t_{i}\right)\right\|}_{2}^{2}}{{\left\|A_{\beta }\left(t_{i}\right)\right\|}_{2}^{2}}+w{\left\|A_{\beta }\left(100;\epsilon_{A_{\beta }}+\theta_{A_{\beta }}^{s}\right)-1\right\|}_{2}^{2}+w_{reg}{\left\|\epsilon_{A_{\beta }}\right\|}_{1},$$
  where $\theta_{A_{\beta }}^{s}=\left(I^{68}D_{A_{\beta }}^{s},I^{68}\lambda_{A_{\beta }}^{s},I^{68}K_{A_{\beta }}^{s}\right)$ and $A_{\beta 0}^{s}$ are the solution of homogenized model of step 1. To avoid overfitting issue, an $L^{1}$ regularization term is imposed for the model parameters. Regularization weight $w_{\text{reg}}$ is tuned to balance fit and sparsity so that to avoid potential overfitting. This optimization problem is solved using MATLAB’s fmincon starting with $\theta_{A_{\beta }}^{s}$ as the initial of the model parameters.
3. **Learnable Initial Conditions:** Extend homogenized initial condition $A_{\beta 0}^{s}$ to non-homogenized vector to account for regional variability, denoted as $A_{\beta 0}^{68D}$. To avoid overfitting issue, an L1 regularization term is imposed for initial condition parameters. Let the sparse deviation from the scalar initial condition denoted as $\epsilon_{A_{\beta 0}}=A_{\beta 0}^{68D}-I^{68}A_{\beta 0}^{s}$. The optimization problem is formulated as:
(14) $$\underset{\epsilon_{A_{\beta 0}}}{\text{min}\;}\sum_{i}\frac{{\left\|A_{\beta }\left(t_{i};\theta_{A_{\beta }}^{68D}\right)-A_{\beta }\left(t_{i}\right)\right\|}_{2}^{2}}{{\left\|A_{\beta }\left(t_{i}\right)\right\|}_{2}^{2}}+w{\left\|A_{\beta }\left(100;\theta_{A_{\beta }}^{68D}\right)-1\right\|}_{2}^{2}+w_{0}{\left\|\epsilon_{A_{\beta 0}}\right\|}_{1},$$
  where $\theta_{A_{\beta }}^{68D}$ is the solution obtained from the previous step and keep fixed during the optimization in this step.
4. **Patient-Specific FC matrix:** For previous 3 steps, population FC matrix is keep fixed. Now, we learn patient-specific FC matrix from PET scan data by incorporating low-rank variation of $L_{p}$, modeled as (10). The constrained optimization problem is formulated as:
(15) $$\underset{u,v}{\text{min}\;}ℒ(u,v)=\sum_{i}\frac{{\left\|A_{\beta }\left(t_{i};\theta_{A_{\beta }}^{68D}\right)-A_{\beta }\left(t_{i}\right)\right\|}_{2}^{2}}{{\left\|A_{\beta }\left(t_{i}\right)\right\|}_{2}^{2}}+w{\left\|A_{\beta }\left(100;\theta_{A_{\beta }}^{68D}\right)-1\right\|}_{2}^{2}+w_{u}{\left(\|u{\|}_{2}-1\right)}^{2}+w_{v}{\left(\|v{\|}_{2}-1\right)}^{2}+w_{su}\left\|u{\|}_{1}+w_{sv}\right\|v{\|}_{1},\;\text{subject}\;\text{to}:\;0\leq a_{kh}+\frac{1}{2}\left(u_{k}v_{h}+v_{k}u_{h}\right)-\delta_{kh}u_{k}v_{h}\leq 1\;\forall k,h=1,\ldots ,68.$$
  where $a_{kh}$ is the kh-th element of FC matrix $A_{p},u=\left(u_{1},\ldots ,u_{68}\right),v=\left(v_{1},\ldots ,v_{68}\right)$ are imposed to ensure stability and interpret ability of the model and optimized by MATLAB fmincon function.

### Homotopy regularization technique for optimization stability

At each optimization step, a regularization term with a corresponding coefficient is introduced to prevent overfitting – a critical requirement for ensuring the learned model’s generalizability. However, directly setting the regularization coefficient to a small target value (often necessary for high model fidelity) leads to training instability due to the complex, non-convex loss landscape. To address this, we propose a homotopy regularization technique inspired by numerical homotopy continuation – a method in computational mathematics that solves hard problems by gradually transforming them from simpler, related problems.

The core idea involves solving a sequence of progressively harder optimization problems through a continuous deformation of the regularization coefficient. Specifically:

1. Initialization: Begin with a large regularization coefficient (e.g., $w_{reg}^{0}=1000$), which simplifies the loss landscape by dominating the objective function, ensuring stable convergence.
2. Progressive Refinement: For each stage, $w_{\text{reg}}^{k},k=0,\ldots ,n$ is fixed and after certain iteration steps of optimizations, one can reduce the the regularization coefficient by a decay factor (e.g., $w_{\text{reg}}^{k+1}=w_{\text{reg}}^{k}/10$). Namely, Gradually reduce the regularization coefficient by a decay factor at each stage.
3. Warm-Start Propagation: Use the solution from the previous stage (larger λ) as the initial guess for the next stage (smaller λ), leveraging the continuity of the homotopy path.

This approach effectively navigates the loss landscape by iteratively transitioning from a heavily regularized, convex-like regime to the target low-regularization regime, mitigating the risk of poor local minima or divergence.

### Sensitivity analysis of model parameters

In this part, we introduce the sensitivity analysis of the model parameters which provides a better understanding of how the changes in the output of a model can be apportioned to different changes in the model parameters [49]. Sobol method [56], a variance-based sensitivity estimates for nonlinear mathematical models, is employed. It obtains the contribution of each parameter to the variance of the quantities of interest (i.e. the model output C(100) in our case). The sensitivity index quantifies a parameter’s influence on the model output—the larger the index, the greater the parameter’s impact, and thus the higher its importance in the model.

Consider a model in the form $Y=f\left(X_{1},\ldots ,X_{k}\right)$ with Y a scalar output of the model, $X_{i}$ is the i–th parameter and $X_{\sim i}$ denotes all parameters but $X_{i}$. The effect on the model output by varying $X_{i}$ alone, called the first-order index of $X_{i}$, is defined as the normalized variance given below

(16) $$S_{1}^{\left(i\right)}=\frac{{\text{Var}}_{X_{i}}\left(E_{X_{\sim i}}\left(Y|X_{i}\right)\right)}{Var(Y)}.$$

The effect on the model output by varying $X_{i}$ and $X_{j}$ simultaneously and removes the effect of their individual first-order indexes, called the second-order index, is defined as

(17) $$S_{2}^{\left(ij\right)}=\frac{{Var}_{X_{i},X_{j}}\left(E_{X_{\sim ij}}\left(Y|X_{i},X_{j}\right)\right)}{Var(Y)}-S_{1}^{\left(i\right)}-S_{1}^{\left(j\right)}.$$

The total index of parameter $X_{i}$ measures the contribution to the output variance of $X_{i}$ including all variance caused by its interactions, of any order, with any other model parameters and it is defined as

(18) $$S_{T}^{\left(i\right)}=\frac{E_{X_{\sim i}}\left[{Var}_{X_{i}}\left(Y|X_{\sim i}\right)\right]}{Var(Y)}=1-\frac{{Var}_{X_{\sim i}}\left[E_{X_{i}}\left(Y|X_{\sim i}\right)\right]}{Var(Y)}.$$

$S_{T}^{\left(i\right)}$ measures the total effects, i.e. first and higher order effects (interactions) of parameter $X_{i}$.

## Figures and Tables

**Figure 7:**
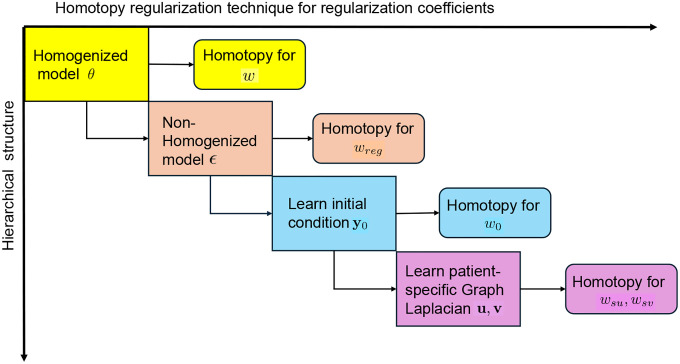
Diagram of the strategies for solving the complex original constrained optimization problem. The original optimization problem is splited into 4 optimization steps with a hierarchical structures as shown vertically. The homotopy reguraluraization technique is applied to every regularization coefficient $w,w_{\text{reg}},w_{0},w_{sv}$, and $w_{su}$ for every optimization step accordingly as shown horizontally.

**Table 1:** Summary reported as (median, mean, std) for each biomarker for homogeneous (H) model and nonhomogeneous (NH) model.

Biomarker	Fit (H) (%)	Test (H) (%)	Fit (NH) (%)	Test (NH) (%)
Abeta	(89.15, 88.87, 1.98)	(87.95, 87.61, 2.87)	(90.78, 91.33, 3.05)	(89.63, 89.48, 3.44)
tau	(91.32, 89.48, 5.57)	(86.71, 85.07, 6.16)	(92.49, 91.36, 5.01)	(88.06, 86.51, 5.62)
N	(81.51, 80.03, 8.18)	(81.38, 79.30, 9.09)	(85.16, 85.49, 5.80)	(83.92, 83.70, 5.82)
C	(95.63, 91.33, 12.31)	(94.98, 88.26, 16.87)	(96.58, 95.80, 3.52)	(95.81, 93.14, 7.59)

**Table 2:** Top DK regions by biomarkers and three disease stages with lobe distribution. Frontal (F): blue, Temporal (T): orange, Parietal (P): green, Limbic (L): purple, Occipital (O): red

Biomarkers	Early Stage (t=60)	Middle Stage (t=80)	Late Stage (t=100)
	24 L	15 P	33 T
$A_{\beta }\left(\lambda_{A_{\beta }}\right)$	68 T	18 T	36 L
	58 T	39 T	18 T
	45 T	36 L	39 T
	55 F	2 L	2 L
	34 P	61 L	61 L
	59 L	6 F	6 F
	35 T	52 T	52 T
	23 O	40 F	40 F
	1 T	27 F	27 F
$A_{\beta }$ **Lobe Count**	F:1, T:5, P:1, O:1, L:2	F:3, T:3, P:1, O:0, L:3	F:3, T:4, P:0, O:0, L:3
	3 F	35 T	39 T
$\tau \left(\lambda_{\tau },K_{\tau }\right)$	64 T	40 F	1 T
	22 T	1 T	52 T
	45 T	52 T	2 L
	59 L	2 L	36 L
	26 L	27 F	27 F
	23 O	18 T	18 T
	25 F	36 L	40 F
	51 P	6 F	6 F
	60 F	61 L	61 L
τ **Lobe Count**	F:3, T:3, P:1, O:1, L:2	F:3, T:4, P:0, O:0, L:3	F:3, T:4, P:0, O:0, L:3
	51 P	48 O	64 T
$N\left(\lambda_{\tau_{N}},\lambda_{N}\right)$	64 T	51 P	4 O
	38 O	26 L	22 T
	22 T	23 O	38 O
	23 O	14 O	6 F
	14 O	64 T	5 T
	4 O	4 O	40 F
	48 O	17 P	39 T
	26 L	22 T	67 T
	59 L	38 O	33 T
N **Lobe Count**	F:0, T:2, P:1, O:5, L:2	F:0, T:2, P:2, O:5, L:1	F:2, T:6, P:0, O:2, L:0
**Total Lobes Count**	F:4, T:10, P:3, O:7, L:6	F:6, T:9, P:3, O:5, L:7	F:8, T:14, P:0, O:2, L:6

**Table 3:** Dataset Table. There are 1891 patients who have at least one of the biomarkers involving at least 3 time points in total. CN: Cognitively Normal, MCI: Mild Cognitive Impairment, AD: Alzheimer’s Disease

	CN	MCI	AD	unknown	Total
${\text{A}}_{\beta }$	142	140	3	186	471
τ (with ${\text{A}}_{\beta }$)	30	36	1	17	84
τ (without ${\text{A}}_{\beta }$)	30	26	16	3	75
N (with ${\text{A}}_{\beta }$ and τ)	40	53	0	14	107
N (with τ only)	19	9	4	0	32
N (with ${\text{A}}_{\beta }$ only)	121	192	1	21	335
N (without ${\text{A}}_{\beta }$ and τ)	87	264	109	20	480
C (with ${\text{A}}_{\beta }$ and τ,N)	40	53	0	14	107
C (with τ,N only)	19	9	4	0	32
C (with ${\text{A}}_{\beta },\text{N}$ only)	121	192	1	21	335
C (without ${\text{A}}_{\beta }$ and τ,N)	83	254	100	20	457

## Data Availability

All multimodal neuroimaging and clinical data used in this study were obtained from the Alzheimer’s Disease Neuroimaging Initiative (ADNI; http://adni.loni.usc.edu/) following approval of our data use application. Regional Aβ-PET and tau-PET standardized uptake value ratios (SUVRs) were derived from the “UC Berkeley - AV45 analysis [ADNI1, GO, 2, 3] (version: 2020-05-12)” and “UC Berkeley - AV1451 analysis [ADNI1, GO, 2, 3] (version: 2022-04-26)” datasets. Cortical thickness measures were obtained from the “UCSF Cross-Sectional FreeSurfer (6.0) [ADNI3]” and “UCSF Cross-Sectional FreeSurfer (5.1) [ADNI1, GO, 2]” releases. The Mini-Mental State Examination (MMSE) scores were obtained from “Mini-Mental State Examination (MMSE) [ADNI1,GO,2,3,4]”. Resting-state fMRI data were collected from 3T scanners following standardized ADNI acquisition protocols, and functional connectivity was computed between DKT-68 atlas regions.

## Code availability

The simulation study code of hierarchical structured training incorporating with homotopy regularization is openly available at https://github.com/chunyanlimath/AD-DigitalTwin-Model. The sensitivity analysis code is available at http://salib.readthedocs.io/en/latest/.
